# Interstitial Flow Recapitulates Gemcitabine Chemoresistance in A 3D Microfluidic Pancreatic Ductal Adenocarcinoma Model by Induction of Multidrug Resistance Proteins

**DOI:** 10.3390/ijms20184647

**Published:** 2019-09-19

**Authors:** Bart Kramer, Luuk de Haan, Marjolein Vermeer, Thomas Olivier, Thomas Hankemeier, Paul Vulto, Jos Joore, Henriëtte L. Lanz

**Affiliations:** 1 Mimetas BV, J.H. Oortweg 19, 2333 CH Leiden, The Netherlands; 2 Leiden Academic Centre for Drug Research, Leiden University, Einsteinweg 55, 2333 CC Leiden, The Netherlands

**Keywords:** pancreatic ductal adenocarcinoma, microfluidics, interstitial flow, gemcitabine, chemoresistance, cancer, modeling

## Abstract

Pancreatic Ductal Adenocarcinoma (PDAC) is one of the most lethal cancers due to a high chemoresistance and poor vascularization, which results in an ineffective systemic therapy. PDAC is characterized by a high intratumoral pressure, which is not captured by current 2D and 3D in vitro models. Here, we demonstrated a 3D microfluidic interstitial flow model to mimic the intratumoral pressure in PDAC. We found that subjecting the S2-028 PDAC cell line to interstitial flow inhibits the proliferation, while maintaining a high viability. We observed increased gemcitabine chemoresistance, with an almost nine-fold higher EC50 as compared to a monolayer culture (31 nM versus 277 nM), and an alleviated expression and function of the multidrug resistance protein (MRP) family. In conclusion, we developed a 3D cell culture modality for studying intratissue pressure and flow that exhibits more predictive capabilities than conventional 2D cell culture and is less time-consuming, and more scalable and accessible than animal models. This increase in microphysiological relevance might support improved efficiency in the drug development pipeline.

## 1. Introduction

The five-year-survival rate for Pancreatic Ductal Adenocarcinoma (PDAC) is as low as 8%, which is the lowest of all solid tumors, and has had little to no change in the past years despite advances in anti-cancer therapy [1]. Due to a lack of early detection methods and few early symptoms, most patients suffering from PDAC are diagnosed at a later stage, when the disease is already advanced. While surgery would be the only curative treatment at this point, only a small number of patients are eligible for surgery, leaving the use of chemotherapeutic compounds as the only option of treatment. The chemotherapeutic compound gemcitabine is widely used to treat various solid cancers, e.g. PDAC. Gemcitabine inhibits DNA synthesis by acting as an analogue of cytidine, preventing chain elongation after its incorporation through blocking of DNA polymerases [2]. Furthermore, gemcitabine is known to induce apoptosis in pancreatic cancer cells via caspase signaling [3,4]. Despite its various cytotoxic effects on pancreatic cancer cells, the effect of gemcitabine on the survival of patients is modest [5], while the side-effects are quite severe. In addition, gemcitabine remains ineffective in treating PDAC due to the characteristics of the disease. In contrast to most solid tumors, PDAC is characterized by a paucity of blood vessels resulting in a systemic therapy being rendered ineffective, due to a lack of delivery of the therapy [6]. Furthermore, the presence of a high intratumoral pressure and a corresponding inordinate interstitial flow from the tumor center towards the tumor periphery, further limits therapeutic efficacy by hampering delivery and is hypothesized to induce a higher resistance to chemotherapy in pancreatic cancer cells. In addition, the patient initially responds well to the treatment, but there is a high occurrence of acquired chemoresistance [7]. Chemoresistance in cancer is linked to the expression of multidrug resistance proteins (MRPs), which are a subfamily of the ATP-binding cassette (ABC) transporters. Indeed, overexpression of MRPs has been observed in gemcitabine-resistant PDAC cell lines, which might explain the acquired chemoresistance in PDAC [8].

The alarming lack of progress in the treatment of PDAC, and concomitantly, the urgent need for a better understanding of the disease, calls for the development and characterization of novel disease model. Up until now, conventional two-dimensional cell culture techniques and animal models have been used to study PDAC. However, a two-dimensional cell culture is known to not fully recapitulate tumor biology due to a lack of physiological relevance [9]. Animal models are expensive, labor-intensive, and suffer from ethical as well as biological limitations [10].

In order to overcome the limitations of traditional disease model systems, organ-on-a-chip systems are gaining widespread interest. [11] Organ-on-a-chip microfluidic systems aim to faithfully recapitulate the physiology and microenvironment of tissues through spatial control of the tissue architecture and the addition of fluid control. These systems typically feature micrometer-sized channels that allow a controlled patterning of extracellular matrices (ECMs) and cells [12]. Organ-on-a-chip systems have been proven to be applicable to a wide variety of tissues, such as vasculature, [13,14] brain, [15] kidney [16], and liver [17].

Recently, we described the use of a high-throughput organ-on-a-chip platform, the OrganoPlate, for therapy response testing of breast cancer, showing the potential of the platform for three-dimensional tumor models and its application in assessing the resistance of cells to chemotherapeutic agents for personalized medicine [18].

Here, we set out to develop and characterize a PDAC three-dimensional cell culture model using the previously mentioned microfluidic platform to assess differences in resistance to chemotherapeutic agents, under interstitial flow. First, we characterized the flow profiles in the platform with the aim to recapitulate the effects of high interstitial fluid pressure in PDAC. Second, we investigated how the different flows affected a non-metastatic pancreatic cancer cell line (S2-028) [19] by assessing the morphology, proliferation, viability, and chemoresistance. Finally, we further characterized the model using gene expression analysis and a functional assay for the MRP family. In our discussion, we presented our view on how organ-on-a-chip tissue models could potentially be used to further our understanding of the PDAC progression and treatment.

## 2. Results

### 2.1. Interstitial Flow Modeling in a Microfluidic Platform

**Figure 1:**
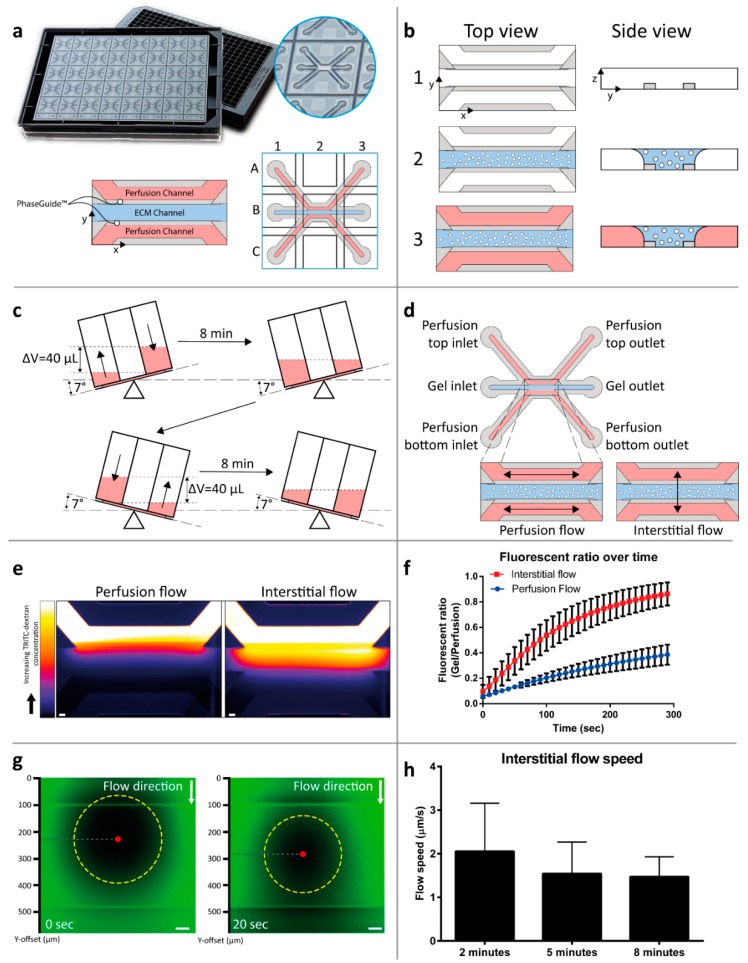
Interstitial flow-on-a-chip characterization. (**a**) The microfluidic microtiter plate ‘OrganoPlate’ was used for a 3D cell culture, based on a 384 well plate interface with 40 microfluidic chips integrated in the bottom. A gel channel (blue) holds the extracellular matrix (ECM) in place through the PhaseGuide’s pressure barrier function. (**b**) Cells can be introduced in the middle lane (gel channel) to create a 3D cell culture with interstitial flow in a collagen ECM. Following the addition of a medium in the adjacent channels (2), the plate is placed on a tilted rocking platform with a rocking interval of 8 min, (**c**) which creates a height difference (equivalent to a 40 µL volume difference) resulting in gravity driven, continuous, and bi-directional perfusion of the cultures. (**d**) The resultant perfusion flow. (**e**) By placing the plate perpendicular to the perfusion flow condition, the flow is directed through the ECM gel in an interstitial flow. The interstitial flow was visualized by introducing a medium volume containing a fluorescent dye coupled to a dextran molecule in the top perfusion channel, thus, creating a pressure difference between the perfusion channels, image is acquired 2 min after introduction of the dye, scale bar = 100 µm). (**f**) Quantification was performed by calculation of the fluorescence ratio in the top perfusion and gel channels (plot represents average ± SD, *n* = 12–15 technical replicates). More detailed interstitial flow measurements within the ECM gel were performed using FRAP. (**g**) Images of the photobleached spot at two consecutive timepoints. The movement of the bleached spot (yellow circle) was tracked by the displacement of its center of mass (red dot). (**h**) The movement of the spot was calculated as an indication of the flow speed over time (*n* = 17 technical replicates, bar plots represent average + SD, scale bar = 50 µm).

Regular two-dimensional tissue cultures do not allow the incorporation of interstitial flow. In order to be able to assess the response of S2-028 PDAC cells to different flow orientations, a three-dimensional tissue model was developed in a microfluidic culture platform. The microfluidic culture platform, the OrganoPlate, is based on a 384-well microtiter plate format. The glass bottom contains 40 microfluidic chips (Figure 1a), with each microfluidic chip positioned under 9 wells in a 3 × 3 grid. A single chip consists of three channels—a center channel (‘gel channel’) that is used to load an extracellular matrix (ECM) and 2 adjacent ‘perfusion channels’, which are loaded with medium. Each channel is connected to 2 wells of the microtiter plate, which act as medium reservoirs. The channels merge together in the center of the chip and three lanes are marked by 2 phaseguides [20], small ridges that form a capillary pressure barrier which confine the ECM precursor mixed with cells to the center lane. Upon gelation, culture media is added to the exterior channels, resulting in a stratified setup without any physical barriers between the channels, such as artificial membranes (Figure 1b). In order to generate a flow parallel to the ECM lane with the PDAC cells, the plate is placed on a tilted rocking platform, creating a height difference and, thus, a pressure difference between the plate wells that are connected to a single channel (‘Perfusion flow’, Figure 1c). In order to generate a flow through the ECM lane with the PDAC cells, the plate is placed on the rocking platform perpendicular to the position in the perfusion flow condition, thus, mimicking interstitial flow Figure 1d). The rocking platform switches between inverted 7° angles of inclination every 8 min to sustain a medium flow over longer periods of time.

To visualize and quantify interstitial flow through the ECM gel, a fluorescent dye, coupled to a 4.4 kDa Tetramethyl Rhodamine Iso-Thiocyanate (TRITC)-dextran, was introduced into the top perfusion channel of a device containing a gelated collagen I ECM in the gel channel. The pressure difference, which is normally generated by placing the plate on a tilted rocker, was simulated by applying a 40 µL volume difference between the top and bottom inlet and outlet. The distribution of the fluorescent signal is shown in Figure 1e. By calculating the ratio over time between the average fluorescent intensity in the ECM gel and in the perfusion channel, a measure for flow in the ECM gel is obtained (Figure 1f). A 3.3-fold faster increase of this ratio was observed for the interstitial flow condition, as compared to the parallel flow condition, indicating a higher flow speed through the ECM gel. To quantify the interstitial flow speed, we utilized the fluorescence recovery after photobleaching (FRAP) method. In short, a localized spot within the gel channel was bleached using a fluorescent light beam and its movement was measured by following the center of mass using imaging and image-processing techniques (Figure 1g). Similar volumes of fluorescent dye-containing solution were applied as in Figure 1e and the flow speed was measured after 2, 5, and 8 min; the latter time interval being the interval of the rocking platform. The average flow speed varied between 1.5 and 2 µm/s (Figure 1h), which is in the range of the interstitial flow speeds found in vivo [21].

### 2.2. Interstitial Flow Inhibits the Proliferation of the PDAC Cells

**Figure 2:**
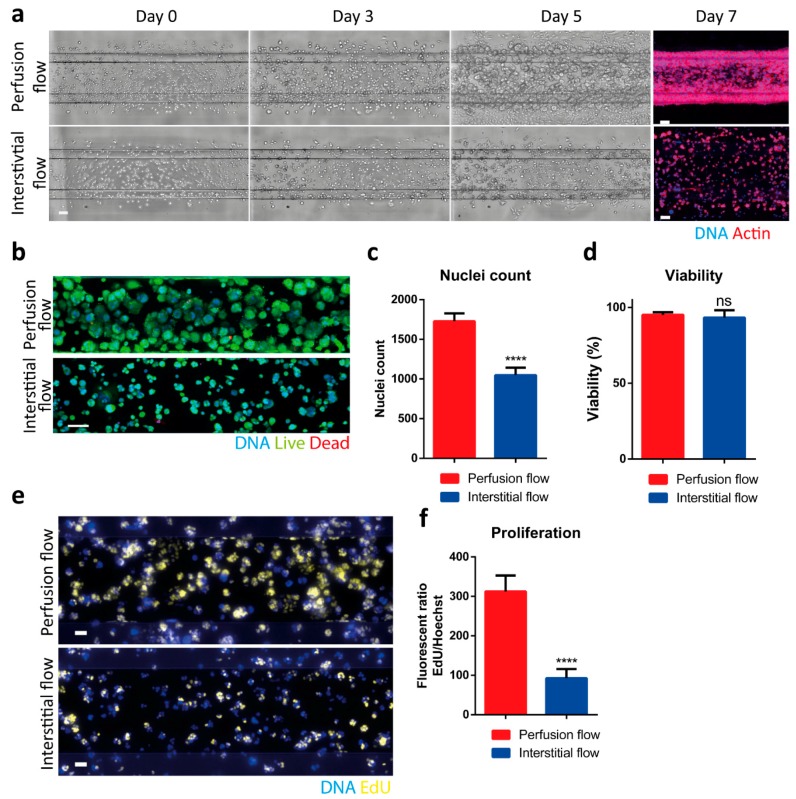
Interstitial flow has pronounced effects on the S2-028 cells. (**a**) A total of 2.5 million S2-028 cells/mL were loaded in the 3-lane OrganoPlate and cultured under different flow conditions; (**right**) immunofluorescent visualization for actin (red) and DNA (Hoechst, blue) of the ECM compartment at day 7. A maximum projection of z-stacks is shown. (**b**) Live (Calcein-AM, green)/dead (propidium Iodide, red) staining at day 5. Representative images are shown for both flow types. (**c**,**d**) Quantification of the number of nuclei and viability (*n* = 10–18 technical replicates). Bar plot represents average + SD. (**e**,**f**) The proliferation rate of the S2-028 cell line was assessed with an EdU (yellow) incorporation assay, determining average EdU fluorescence, normalized for the amount of DNA using the Hoechst (blue) signal (*n* = 14 technical replicates, bar plot represents average + SD). Scale bar in Figure **a**–**c** is 100 µm, **e** is 50 µm. Statistical test: unpaired two-tailed t-test (ns = not significant, **** *p* < 0.0001).

In order to study the influence of interstitial flow on pancreatic cancer cells, the non-metastatic PDAC cell-line S2-028 was loaded in collagen ECM and subjected to either the Perfusion or Interstitial flow profile (Figure 1d). Rapid proliferation and spheroid formation were observed when the cells were subjected to perfusion flow (Figure 2a, top row). When the cells in ECM were subjected to interstitial flow, smaller spheroids were observed from day 3 onwards (Figure 2a, bottom row). Immunofluorescent staining of the PDAC cells in the ECM channel at day 7 confirmed this observation (Figure 2a, right). Figure 2a also shows that cells cultured with perfusion flow seemed to contract the ECM, in contrast to cells cultured with interstitial flow. Immunofluorescent viability staining for live (Calcein-AM) and dead (Propidium Iodide) cells (Figure 2b) showed a decreased cell count in the interstitial flow condition (Figure 2c), whereas no difference was observed in the overall viability of cells (Figure 2d), suggesting a decreased proliferation rate as a result of interstitial flow. Similarly, the PDAC cells showed a lower EdU incorporation under interstitial flow, as compared to the perfusion flow condition, also suggesting a decreased proliferation rate (Figure 2e,f).

### 2.3. PDAC Cells are More Resistant to Gemcitabine in the Presence of Interstitial Flow

**Figure 3:**
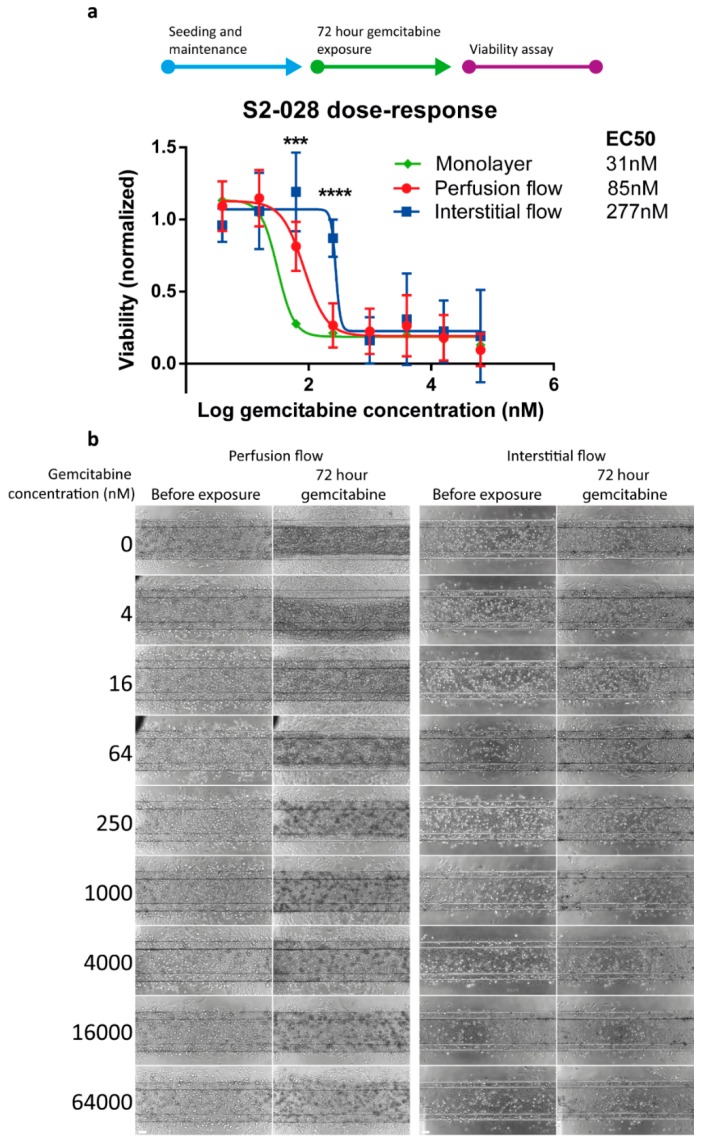
PDAC cells cultured with interstitial flow are less responsive to gemcitabine. S2-028 cultures were exposed to a dose-range of gemcitabine for 72 h. (**a**) Dose-response curves for the two-dimensional monolayer and both flow models with the EC50 values were extracted from these curves. *N* = 3 independent experiments for both 3D models and *N* = 2 for the monolayer with *n* = 4–11 technical replicates for each iteration. Shown is the average value normalized to the vehicle control ± SD. Statistical test—multiple two-tailed *t*-test; showing significant difference for perfusion flow versus interstitial flow *** *p* < 0.001, **** *p* < 0.0001. (**b**) Phase contrast images of the S2-028 cell line after 72 h gemcitabine exposure under perfusion or interstitial flow. Decrease in cell density and cell death can be observed in the perfusion flow condition, upwards from 64 nM gemcitabine. When S2-028 cells are cultured under interstitial flow, these effects are observed upwards from 250 nM gemcitabine. Scale bar = 100 µm.

The chemotherapeutic compound gemcitabine remains a cornerstone of PDAC treatment in all stages of the disease, although its contribution to survival is negligible due to resistance of the tumor [5]. Particularly in PDAC, the tumor microenvironment plays a crucial role in therapy resistance with a suggested role of matrix deposition blocking the delivery of the chemotherapeutic agent to the tumor cells by increasing the interstitial fluid pressure [22]. Gemcitabine acts as an analogue of cytidine and its incorporation results in chain termination. Furthermore, gemcitabine enhances its own activity by depleting the intracellular pool of cytosine triphosphates and induces apoptosis through caspase signaling. To assess the effect of different flow directions on the cytotoxic effect of gemcitabine, 72 h dose response curves of gemcitabine were obtained. The results showed an EC50 of 85 nM and 277 nM for the perfusion and interstitial flow models, respectively, representing a 3-fold lower sensitivity with interstitial flow (Figure 3). When compared to the 31 nM EC50 in 2D monolayers, EC50 values were almost 3- and 9-fold higher for perfusion and interstitial flow in 3D cultures, respectively. The representative phase contrast images of the S2-028 cultures after 72-h exposure to a concentration range of gemcitabine can be found in Figure 3b.

### 2.4. MRP Protein Function is Altered under Interstitial Flow

**Figure 4:**
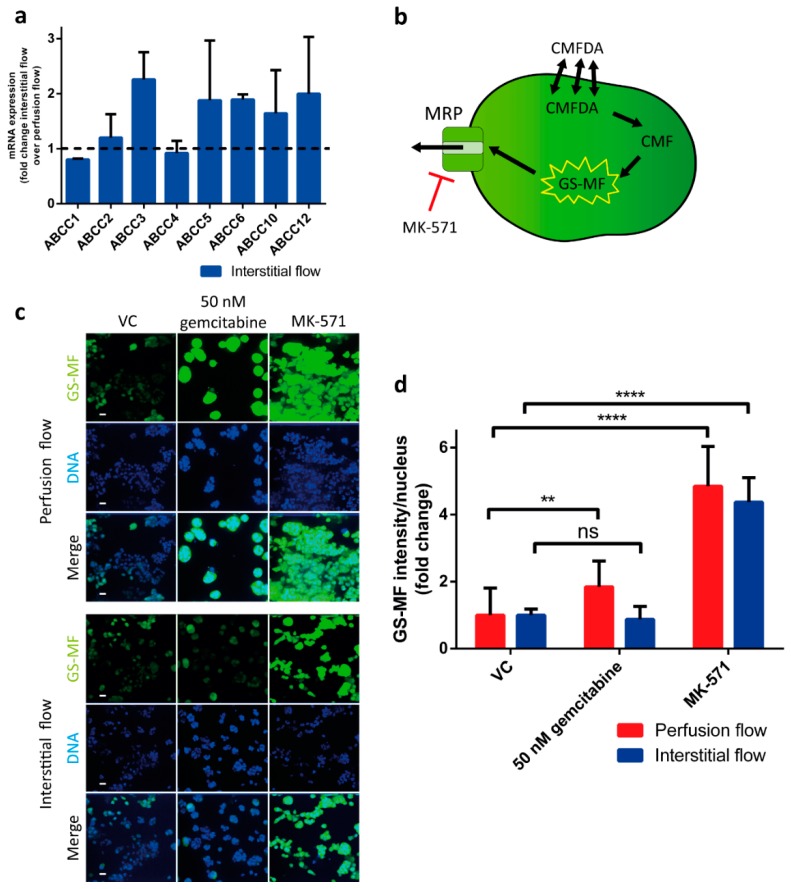
Multidrug resistance protein (MRP) function is increased by interstitial flow. **(a)** mRNA expression levels in S2-028 cells for 8 *ABCC* genes. Gene expression levels were normalized to the reference gene, *TBP*, and a fold change difference, compared to the S2-028 cultures under perfusion flow, was calculated (*N* = 2 independent experiments, *n* = 2 technical replicates, bar plot represents average + SD). **(b)** Principle of the MRP efflux assay. Black arrows indicate a reaction or transportation, red t arrow indicates an inhibitory effect. **(c)** Representative images of the intracellular accumulation of GS-MF (green) in S2-028 cells (scalebar = 50 µm). Nuclei are stained in blue (Hoechst). **(d)** Quantification of the fold change of the average fluorescent intensity per nucleus of the fluorescent GS-MF signal. Values were normalized to vehicle control (VC) per experiment (*N* = 2 independent experiments, *n* = 7–8 technical replicates). Statistical test—unpaired two-tailed t-test (ns = not significant, ** *p* < 0.01, **** *p* < 0.0001).

Development of tumor resistance to gemcitabine critically limits the efficacy of this cornerstone treatment of pancreatic cancer. [5] One class of transporters associated with cancer resistance is the multidrug resistance protein (MRP) family [23]. To investigate whether our 3D culture flow models can be used to assess mechanisms of acquired chemoresistance, the models were subjected to a gene expression analysis of MRP transporters and a functional MRP assay. Gene expression analysis revealed that 5 out of 8 MRP genes showed elevated mRNA expression levels in interstitial flow, as compared to perfusion flow (Figure 4a). A functional CMFDA efflux assay was performed to evaluate the effects of perfusion and interstitial flow models on the activity of MRP transporters in the presence of gemcitabine. The principle of the assay is depicted in Figure 4b. In short, CMFDA, a compound that can freely pass through cell membranes, is metabolized to 5-chloromethylfluorescein (CMF) and then further metabolized to carboxylfluorescein-glutathione (GS-MF, a fluorescent cell membrane impermeant compound). GS-MF is a substrate for MRPs that transport the compound out of the cell, therefore, its fluorescent signal is inversely related to the MRP activity [24]. In cultures subjected to interstitial flow, exposure to 50 µM gemcitabine yielded no significant change in MRP activity, whereas perfusion flow resulted in a significant (1.8-fold) increase in the GS-MF signal, suggesting a decrease in the MRP activity (Figure 4c–d). No significant difference was observed between the perfusion flow and the interstitial flow 3D models when MRP transporters were blocked with the pharmacological inhibitor MK-571 (4.8- and 4.3-fold, respectively). Our results strongly suggest that in these models, interstitial flow contributes to gemcitabine resistance by increasing its inherent activation of efflux pumps.

## 3. Discussion

A substantial amount of cancer research aimed at finding new targets and preclinical drug testing is performed in conventional 2D cell culture and animal models. Typically, 2D cell culture models offer a higher throughput and lower cost, but are less physiologically relevant and are known to be unpredictive [25]. Animal models, on the other hand, offer tissue environments that are more representative of the human body, but have various biological and ethical limitations, are expensive and labor intensive and often lack predictivity [9].

To alleviate the need for better models for cancer research, scientists increasingly embrace 3D tissue culture in ECMs. The culturing of cells in 3D is known to be biologically more relevant, as compared to conventional 2D cell culture [26]. For example, it has previously been found that cultures of breast cancer cells respond differently to chemotherapeutic compounds when cultured in 3D compared to 2D, most likely better reflecting in vivo efficacies in patients [18].

PDAC is characterized by a high intratumoral fluid pressure, a characteristic which is not captured by the existing in vitro PDAC models. In this report, we describe a novel microfluidic 3D cell culture approach to develop and characterize interstitial flow models for PDAC to mimic intratumoural fluid pressure, using the pancreatic cancer cell line S2-028 embedded in an ECM gel. We observed increased chemoresistance of S2-028 cells cultured in 3D compared to monolayer cultures. Interestingly, subjecting 3D cultures of S2-028 cells to a flow profile mimicking interstitial flow in PDAC had pronounced effects on cell morphology and proliferation, without affecting viability. Furthermore, we observed that the EC50 value for gemcitabine increases threefold when tested on cells cultured in 3D as compared to cells cultured as a monolayer, which is closer to the plasma concentration of gemcitabine found in PDAC patients [27]. Thus, the development of novel 3D models that capture specific characteristics of PDAC are likely to spur the development of novel therapies against this disease.

Since gemcitabine acts as an analogue of cytidine and exerts its cytotoxic effect by being incorporated into the DNA of a replicating cell, it stands to reason that slower proliferating cells are affected less by this chemotherapeutic compound. However, it seems unlikely that slower proliferation singularly accounts for the significant increase in chemoresistance observed, as gemcitabine is known to have multiple cytotoxic effects on target cells, e.g., induction of apoptosis via caspase signaling [28]. In addition, Hagmann et al. observed an increased expression of multidrug resistance protein 5 (MRP5) in gemcitabine resistant pancreatic cancer cell line [29]. To investigate this potential, additional mechanism for the increased resistance to gemcitabine with the interstitial flow model, we focused on the expression and function of the family of MRP transporters. In these experiments, we observed a marked increase in the mRNA expression of 5 MRPs when PDAC cells were subjected to interstitial flow. These results strongly suggest that flow-induced MRP expression contributes to our observed increase in chemoresistance by elevating efflux transport of the drug [30,31,32]. This notion is further substantiated by our observation that interstitial flow appears to neutralize an inherent deactivation of MRP activity by gemcitabine, which we observed under perfusion flow. We hypothesize that the apparent increase in MRP activity is caused by a further increase of MRP expression after exposure to gemcitabine under interstitial flow (Figure S1). Although these preliminary results need further investigation, they hint towards an interesting additional role of interstitial flow in establishing gemcitabine resistance. Although the family of MRPs are known to be involved in the acquisition of chemoresistance by cancer cells, the mechanism underlying this process remains enigmatic. Promoter methylation of the ABCC genes is possibly involved, however, these studies have been performed using monolayers of pancreatic cancer cells and should be verified using more relevant three dimensional models [33].

Interstitial pressure and associated interstitial flow, being hallmarks of PDAC, are bound to have other profound effects on the cancer cells that contribute to PDAC pathogenesis, in addition to their effects on MRP function and expression. For example, interstitial flow has been linked to the migratory behavior of cancer cells in biological, as well as mathematical models [34]. These results suggest that there are competing tumor cell migration mechanisms that occur due to interstitial flow, a CCR7-dependent mechanism that induces downstream migration and a CCR7-independent mechanism that promotes cells to migrate upstream. Although flow in our model is bidirectional due to the utilization of a pump-free rocker system, it would be of interest to test whether expression of genes previously implicated to affect migration in cancer, like EPCAM and integrin-β4 [35] is affected in our interstitial flow model.

In conclusion, we developed the basis for a 3D cell culture model for PDAC using a microfluidic platform. We have demonstrated the effects of interstitial flow on the drug response of perfused 3D cell cultures of S2-028 cells, a non-metastatic pancreatic cancer cell line. While our current model does not yet fully capture the in vivo complexity of PDAC, it likely exhibits higher predictive capabilities than conventional 2D cell culture and is less time-consuming, more scalable, and more accessible in comparison to animal models. Drug screening on monolayers is very likely to result in an overestimation of the effects of chemotherapeutics, as is evident from our study. Furthermore, we showed that our model is amenable to interrogation by imaging, functional assays, and gene expression analysis. Finally, containing as much as 40 chips on a microplate footprint, the OrganoPlate offers a high-throughput platform for predictive drug testing and could potentially even be used for personalized therapy selection. Thus, we strongly believe that the models presented here point the way towards valuable tools for the search for novel therapies against PDAC.

## 4. Materials and Methods

### 4.1. Cell Culture

The S2-028 Pancreatic Ductal AdenoCarcinoma cell line (kind gift from Dr. Buchholz, Marburg University) was cultured in T-75 flasks (Corning 431464U, Corning, NY, USA) in DMEM (Sigma D6546, St. Louis, MO, USA) culture medium supplemented with 10% heat inactivated Fetal Bovine Serum (FBS, Gibco 16140-071, Waltham, MA, USA) and 1% Penicillin-Streptomycin (Sigma P4333). S2-028 were used between passage number +7 till +11.

### 4.2. OrganoPlate Culture

The three-lane OrganoPlate with 400 µm × 220 µm (*w* × *h*) channels (MIMETAS 4003-400B, Leiden, The Netherlands) was used to set up the three-dimensional PDAC model. OrganoPlate ECM loading and cell seeding protocol and PhaseGuide functioning was previously described in [36]; the gel and perfusion channel lengths were 9 mm and 12.2 mm, respectively. In short, the protocol is as follows—before seeding, 50 µL of Hank’s balanced salt solution (HBSS) was dispensed into the observation window to prevent evaporation and enhance optical clarity (Figure 1a). S2-028 were trypsinized using 0.25% trypsin in phosphate-buffered saline/ethylenediaminetetraacetic acid (Gibco 15090-046, Waltham, MA, USA) and resuspended in the appropriate volume (2.5 × 10^6^ cells/mL) of ECM gel composed of 9 mg/mL rat tail collagen I (Corning 354249, Corning, NY, USA), 100 mM HEPES (Gibco 15630-056), and 3.7 mg/mL Na_2_HCO_3_ (Sigma-Aldrich S5761, St. Louis, MO, USA). The final concentration of collagen I was 7.2 mg/mL. Two microliters of the ECM-cell suspension was dispensed in the gel inlet and incubated for 30 min at 37 °C, allowing gelation of the ECM (Figure 1b). After gelation of the ECM, 50 µL of DMEM 10% FCS medium was dispensed in the perfusion inlets and outlets. Subsequently, the plate was placed in the incubator (37 °C, 5% CO_2_) on a rocking platform (8 min interval at an angle of 7°, Figure 1c). For the interstitial flow condition, the plate was placed perpendicular to the perfusion flow condition, to introduce a flow through the gel channel (Figure 1d). The medium was changed three times per week.

### 4.3. Interstitial Flow Simulation

To study the flow profile of the interstitial flow condition, a 7.2 mg/mL collagen I gel (without cells) was loaded in the 3-lane OrganoPlate as described above. The perfusion inlet and outlets were filled with 50 µL of HBSS to prevent the channels from drying out. After 24 h incubation in the incubator (37 °C, 5% CO_2_), ECM filled chips were subjected to interstitial flow by pipetting a 40 µL volume difference (60 µL in the top in- and outlet of 0.5 mg/mL TRITC-Dextran 4.4 kDa (Sigma-Aldrich T1037) in HBSS, 40 µL HBSS in the bottom in- and outlet). This recreated the fluid pressure in the microfluidic chip, comparable to placing an OrganoPlate^®^ on a 7° rocking platform in the interstitial flow orientation (Figure 1c). Chips with equal volume (50 µL in the perfusion in- and outlets) were used to mimic the perfusion flow condition. Directly after applying the volume difference, a time lapse series of images were captured (interval 10 s for 3 min) on a Molecular Devices ImageXPress XLS fluorescent microscope (Molecular Devices, San Jose, CA, USA). Images acquired were analyzed using Fiji (version 2, build 1.52e (open source software) [37]. For the visualization of images, a lookup table was applied to map the color scale. For quantification, regions of interest (ROIs) of the perfusion channel and the gel channel were manually drawn and the average fluorescent intensity was measured. By dividing the intensity of the gel channel by the intensity of the perfusion channel the fluorescent ratio was calculated.

Interstitial flow was further characterized using the fluorescent recovery after the photobleaching (FRAP) method. After 24 h of incubation in the incubator (37 °C, 5% CO_2_), the HBSS was aspirated from the 7.2 mg/mL collagen I gel and a volume difference was created (60 µL in the top inlet and outlet, 40 µL in the bottom inlet and outlet) using 2.5 ng/mL fluorescein (Sigma 46960) in HBSS to mimic the interstitial flow condition. After 2, 5, and 8 min, a spot in the middle of the gel channel was bleached with the Molecular Devices ImageXPress Micro Confocal High-Content Imaging System (Molecular Devices, San Jose, CA, USA) for 5 s with 60× magnification. Subsequently a time-series of images was captured (2 s interval for 20 s). Images were processed using Fiji (version 2, build 1.52e) by creating a threshold image of the bleached area. The center of mass was calculated with the ‘analyze particles’ option (size, 1000-infinity). For each image, the shift in the center of mass per second was calculated from the original bleached image.

### 4.4. Live/dead Assay

Viability of the cells was assessed at day 7 of culture in the OrganoPlate. The Medium was aspirated from the cultures and replaced with a mixture of NucBlue (2 drops/mL, Life Technologies R37610, Waltham, MA, USA), Propidium Iodide (PI, 2 drops/mL, Life Technologies R37610, Waltham, MA, USA) and 0.5 µg/mL Calcein-AM (25 µL in each perfusion inlet and outlet, Thermo Fisher Scientific C3099, Waltham, MA, USA). The cultures were incubated on a rocking platform (8-min interval at an angle of 7°) for 1 h in the incubator (37 °C, 5% CO_2_). Subsequently, the culture was imaged on a Molecular Devices ImageXPress XLS fluorescent microscope. Images acquired were analyzed using Fiji (version 2, build 1.52e). The number of nuclei was extracted using an approach based on morphological shape filtering using built-in tools available in Fiji. Nuclei were extracted by removing the background signal via a Rolling Ball method [38]. Afterwards, a threshold was applied to the remaining signal to highlight the nuclei, and a particle detection was subsequently performed to count the number of nuclei. A similar approach was used to quantify the number of PI positive cells, after which the viability was calculated by calculating the ratio of live cells (total nuclei minus PI positive cells) to the total cell count.

### 4.5. EdU Proliferation Assay

The proliferation rate of day 3 cultures was assessed with the EdU Click-iT Plus assay (Thermo Fischer C10640, Waltham, MA, USA) according to manufacturer’s protocol. Culture medium was replaced with a 50 µM dilution of EdU in a culture medium for 24 h, after which the cultures were fixed with 3.7% formaldehyde (Sigma-Aldrich 252549, St. Louis, MO, USA) in HBSS for 10 min, washed twice with HBSS for 5 min, and permeabilized with 0.3% Triton ×-100 (Sigma-Aldrich T8787) in HBSS for 10 min, after which the HBSS was aspirated. The proliferating cells were visualized by adding 20 µL of Click-iT Plus reaction cocktail for 30 min. Subsequently all DNA was visualized by adding 5 µg/mL Hoechst 33342 (Thermo Fisher H3570, Waltham, MA, USA) for 2 h. Z-series images were captured on the Molecular Devices ImageXPress Micro Confocal High-Content Imaging System and the summarized intensity-projections were saved for quantification. Images were analyzed using Fiji (version 2, build 1.52e) by measuring the average fluorescent intensity of the gel channel, after background correction.

### 4.6. Immunohistochemistry

Cultures were fixed using 3.7% formaldehyde (Sigma-Aldrich 252549) in HBSS (Sigma-Aldrich H6648) for 20 min, washed twice with HBSS for 5 min, and permeabilized with 0.3% Triton X-100 (Sigma-Aldrich T8787) in HBSS for 10 min. After washing with 4% FBS in HBSS for 5 min, the cultures were incubated for 60 min with a mixture of ActinRed 555 Readyprobes (2 drops/mL, Thermo Fisher R37112) and 5 µg/mL Hoechst 33342 (Invitrogen H3570, Waltham, MA, USA). Z-series images were captured on the Molecuar Devices ImageXpress Micro Confocal High-Content Imaging System(Molecular Devices, San Jose, CA, USA) and the maximum intensity-projections were used for further representation.

### 4.7. Drug Exposure and Viability Assessment

On the third day, three-dimensional cultures were exposed to the chemotherapeutic compound gemcitabine (Sigma G6423) in a concentration range (4–64.000 nM) for 72 h in the incubator (37 °C, 5% CO_2_) on a rocking platform (8-min interval at an angle of 7°), in both perfusion settings (Figure 1d). For the two-dimensional culture exposure, the cells were grown in a 96-well plate (Corning) for 1 day until 50% confluency and were exposed to the same concentration range of gemcitabine for 72 h in the incubator (37 °C, 5% CO_2_).

### 4.8. Enzymatic Activity Assessment

The enzymatic activity of the cultures after treatment was determined using the WST-8 viability assay. The culture medium in the OrganoPlate was replaced with 25 µL of WST-8 reagent (Sigma-Aldrich 96992) diluted 1:11 in HBBS in each perfusion inlet and outlet. The cultures were incubated for 2 h in the incubator (37 °C, 5% CO_2_) on a rocking platform (8-min interval at an angle of 7°), and the absorbance was measured at 450 nm using the Fluoroskan Ascent plate reader (Thermo Scientific 5210470). The measurements of the gel inlet, perfusion inlet and outlet, and observation window were adjusted for volume differences and were combined. Data were normalized against the vehicle control and plotted in Prism (GraphPad Software version 6). The nonlinear regression analysis ’log(inhibitor) versus response minus Variable slope (four parameters)’ was performed to obtain the half maximal effective concentration (EC50) values.

### 4.9. qPCR

To assess the mRNA levels, S2-028 OrganoPlate cultures were exposed to gemcitabine (Sigma-Aldrich G6423) at day 3 for 72 h. Cells were lysed and RNA was purified using the TRIzol reagent (Thermo Fisher 15596026) with 7 µg Rnase-free glycogen (Thermo Fisher R0551) per sample added as a carrier, according to manufacturer’s protocol. Four to twenty chips were pooled into 1 sample, depending on the condition to compensate for the difference in cell density between the conditions. RNA concentration was measured using a NanoDrop (Thermo Fisher) and the samples were diluted to 30 ng/µL with RNase-free water. cDNA synthesis was performed using M-MLV reverse transcriptase (Thermo Fisher 28025013), according to manufacturer’s protocol. qPCR was performed using the FastStart Essential DNA Green (Roche 06402682001, Rotkreuz, Switzerland) using specific primers for the different MRP genes and using TBP as the housekeeping gene, see Table S1. The data were analyzed using the Roche LightCycler software version 1.1 and the 2^−ΔΔCt^ method, normalizing all values to the perfusion flow vehicle control per experiment.

### 4.10. MRP Efflux Assay

MRP transport in the microfluidic platform was measured as previously described [39]. Day 3 OrganoPlate cultures were exposed to 0 or 50 nM gemcitabine. After 72 h of drug exposure, the medium was replaced with 25 µL 1.25 µM Cell tracker reagent 5-choloromethylfluorescein diacetate (CMFDA, Invitrogen C7025, Waltham, MA, USA) in Opti-HBSS (1:2 Opti-MEM (Gibco 11058-021) and HBSS) in all perfusion inlets and outlets, with and without inhibitor (50 µM MK-571 (Sigma-Aldrich M7571)). The culture was incubated for 30 min in the incubator (37 °C, 5% CO_2_) on a rocking platform (8-min interval at an angle of 7° in the perfusion flow orientation), after which the observation window reservoir was used to cool down the culture with 50 µl 4 °C HBSS. The in-chip assay solutions were replaced by 50 µl of 4 °C inhibition cocktail (10 µM MK-571, 10 µM Ko143 (Sigma-Aldrich K2144) and 5 µg/mL Hoechst 33342). The inhibition cocktail was incubated for 30 min at RT. Z-slices were imaged using the ImageXPress Micro Confocal High-Content Imaging System (Molecular Devices). The data were analyzed using Fiji to calculate the intensity of the sum-projection of the z-slices of the FITC channel for the amount of CMFDA. The number of nuclei was obtained in the same manner as described in the live/dead assay section. Relative intensity was calculated by first subtracting the background intensity from the measured intensity and subsequently dividing by the number of cells. Statistics were done with the ‘multiple 2-tailed *t*-tests’ function in GraphPad Prism version 6.

## Supplementary Materials

Supplementary materials can be found at https://www.mdpi.com/1422-0067/20/18/4647/s1.

## Abbreviations

MRP	multidrug resistance protein
PDAC	PancreaticDuctal Adenocarcinoma
ABC	ATP-binding cassette
ECM	extracellular matrix
FRAP	Fluorescence Recovery After Photobleaching
CMF	5-chloromethylfluorescein
CMFDA	5-choloromethylfluorescein diacetate
GS-MF	carboxylfluorescein-glutathione
TRITC	Tetramethyl Rhodamine Iso-Thiocyanate
