# Supplementary material for: Interstitial Flow Recapitulates Gemcitabine Chemoresistance in A 3D Microfluidic Pancreatic Ductal Adenocarcinoma Model by Induction of Multidrug Resistance Proteins
Source: Int J Mol Sci. 2019 Sep 19;20(18):4647. doi: 10.3390/ijms20184647 (PMC6770899; doi:10.3390/ijms20184647)
Supplement: Supplementary file 1 [file ijms-20-04647-s001.pdf]

International Journal of
Molecular Sciences

Supplementary:

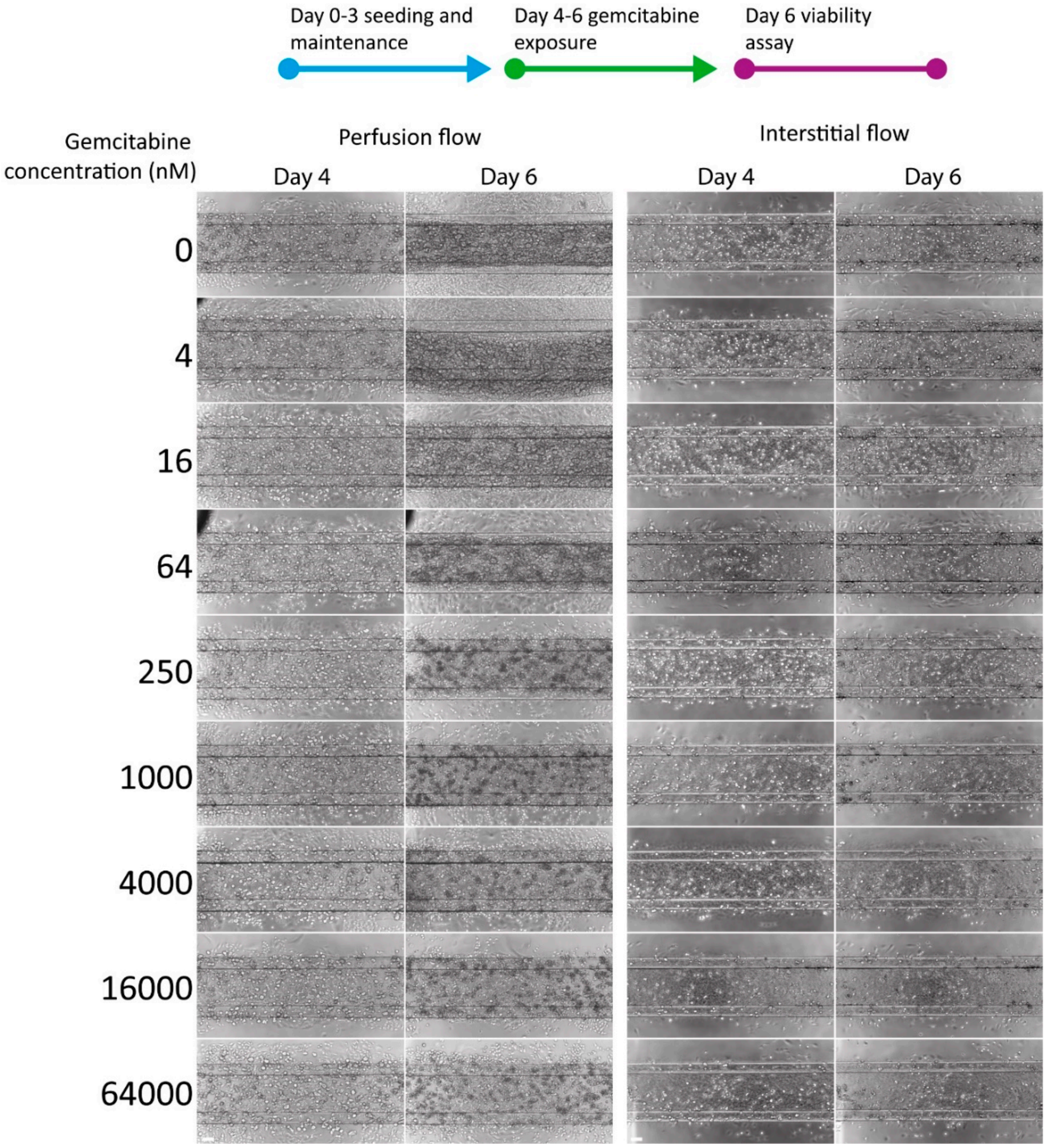

Supplementary Figure 1. Phase contrast images of S2-028 cell line after 72 h gemcitabine exposure under perfusion or interstitial flow. Decrease in cell density and cell death can be observed in the perfusion flow condition upwards from 64 nM gemcitabine. When S2-028 cells are cultured under interstitial flow these effects are observed upwards from 250 nM gemcitabine. Scaleb bar = 100 μm

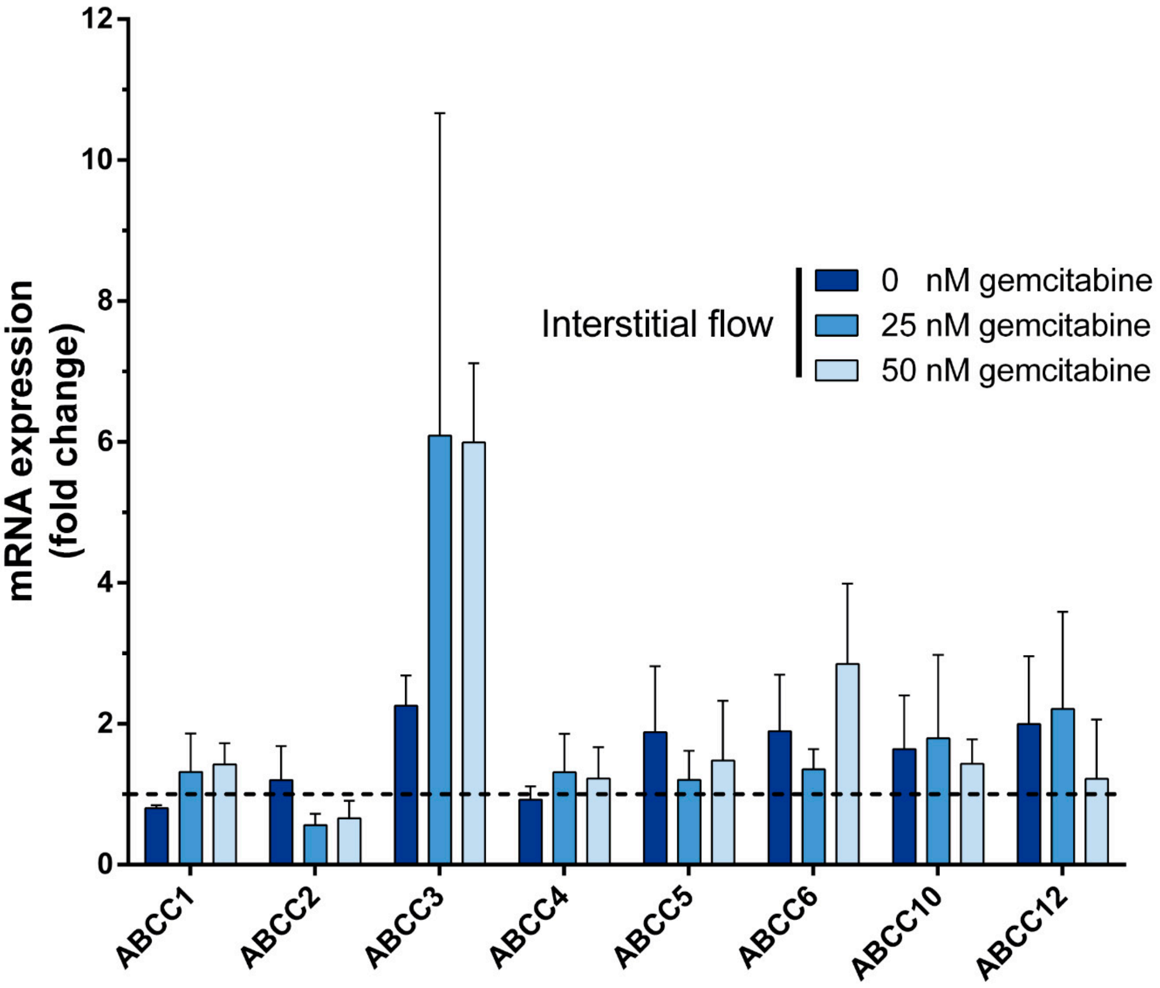

Supplementary Figure 2 Fold change of mRNA expression levels of the ABCC genes after 72-hour exposure of S2-028 to gemcitabine. Gene expression levels were normalized to the reference gene TBP. Fold changes were calculated per experiment for interstitial flow compared to perfusion flow for each concentration of gemcitabine (*N* = 2 independent experiments, *n* = 2 technical replicates).

Supplementary table 1. Primer sequences used for qPCR.

**Gene**	**Forward primer**	**Reverse primer**
TBP	TGCACAGGAGCCAAGAGTGAA	CACATCACAGCTCCCCACCA
ABCC1	GCCGAAGGAGAGATCATC	AACCCGAAAACAAAACAGG
ABCC2	AGAGCTGGCCCTTGTACTCA	AGGGACAGGAACCAGGAGTT
ABCC3	GTGGGGATCAGACAGAGAT	TATCGGCATCACTGTAAACA
ABCC4	TGTTTGATGCACACCAGGAT	GACAAACATGGCACAGATGG
ABCC5	CAGCCAGTCCTCACATCA	GAAGCCCTCTTGTCTTTTTT
ABCC6	AGGAGGCCCGAGCTTAGAC	CCTGCCGTATTGGATGCTGT
ABCC10	GTCCAGATTACATCCTACCCTGC	GCCAACACCTCTAGCCCTATG
ABCC12	ATGCGGTTGTCACTGAAG	GTTGCCTCATCCATAATAAGAAT
